# Sustained expression of inflammatory monocytes and activated T cells in COVID‐19 patients and recovered convalescent plasma donors

**DOI:** 10.1002/iid3.476

**Published:** 2021-08-06

**Authors:** Ravinder Singh, Hamed Hemati, Meenu Bajpai, Pushpa Yadav, Ashish Maheshwari, Suresh Kumar, Sonal Agrawal, Jayesh Kumar Sevak, Mojahidul Islam, Jaswinder Singh Mars, Shiv K Sarin, Nirupama Trehanpati

**Affiliations:** ^1^ Laboratory of Molecular Immunology, Department of Molecular and Cellular Medicine Institute of Liver and Biliary Sciences New Delhi India; ^2^ Department of Transfusion Medicine Institute of Liver and Biliary Sciences New Delhi India; ^3^ Department of Medicine Lok Nayak Jai Prakash Hospital New Delhi India; ^4^ Department of Hepatology Institute of Liver and Biliary Sciences New Delhi India

**Keywords:** convalescent plasma, COVID‐19, cytokines, IL‐6, immune cells, MIP

## Abstract

**Introduction:**

Intense monocyte activation and infiltration into the target tissues are the main mechanisms of lung injury in severe acute respiratory syndrome coronavirus 2 infection. A reduction in the degree and nature of such cellular responses is expected following recovery. We aimed to investigate the immune responses in moderate coronavirus disease 2019 (COVID‐19) patients and recovered patients.

**Methods:**

Moderate COVID‐19 patients (*n* = 34) at Lok Nayak Hospital, New Delhi, and COVID‐19 recovered patients (*n* = 15) from the mild disease who were considered for convalescent plasma (COPLA) donation at the Institute of Liver and Biliary Sciences, New Delhi and healthy individuals (*n* = 10), were recruited. We have assessed 21 plasma cytokines using cytokine bead array, performed proteomics on serum proteins, and analyzed immune cells using a detailed multicolor flow cytometry.

**Results:**

A significant increase in inflammatory markers such as macrophage inflammatory protein (MIP)1‐α, monocyte chemotactic protein‐1, macrophage migration inhibitory factor, vascular endothelial growth factor‐A, and Leptin was observed in the moderate patients. Nonsurvivors additionally showed increased interleukin (IL)‐6 levels. Consistently, the proteomics analysis showed the signatures of cytokine production and interferon‐γ response, and increased level of acute‐phase protein SAA1 in the serum of COVID‐19 patients. Despite the sustained expression of MIPs, the recovered COPLA donors showed a surge in MCSF and IL‐18 levels. Both the groups had increased CCR2, CX3CR1 positive monocytes, low CD8^+^ T cells, A proliferation‐inducing ligand, and B‐cell activating factor receptor^+^ B cells compared with healthy subjects.

**Conclusions:**

Patients who have recovered and considered for COPLA donations still have compromised immunity with sustained expression of inflammatory monocytes and activated T cells.

## Graphical abstract

Covid patients who have recovered and considered for considered for convalescent plasma donations still have compromised immunity with sustained expression of inflammatory monocytes and activated T cells

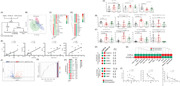

**Abbreviations**
APRIL: A proliferation‐inducing ligand
BAFFR: B‐cell activating factor receptor
BMI: body mass index
CBC: complete blood count
COPD: chronic obstructive pulmonary disease
COPLA: convalescent plasma
DC: dendritic cells
ENA78: neutrophil‐activating peptide
FiO_2_
: fraction of inspired oxygen
HC: healthy controls
HIV: human immunodeficiency virus
IFN: interferon
IL: interleukins
IP‐10: IFN‐γ inducible protein 10
ITAC: interferon‐inducible T‐cell α chemoattractant
MCP: monocyte chemoattractant protein
MIP: macrophage inflammatory protein
MMP: matrix metalloproteinase
PaO_2_
: partial pressure of oxygen
PBMC: peripheral blood mononuclear cells
PCR: polymerase chain reaction
RR: respiratory rate
*SD*
: standard deviation
SOFA score: sequential organ failure assessment score
TGF‐β: transforming growth factor b
Tregs: regulatory T cells
VEGF: vascular endothelial growth factor
WB: whole blood

## 1 INTRODUCTION

In December 2019, a new member of the Coronavirus family emerged in Wuhan, China; later termed as severe acute respiratory syndrome coronavirus 2 (SARS‐CoV‐2) causing a respiratory illness.^1^, ^2^, ^3^ SARS‐CoV‐2 eventually spread across the globe causing over 147.7 million infections and more than 3.1 million deaths. In India, SARS‐CoV‐2 has infected around 17.5 million people and has claimed around 200 thousand lives (https://www.worldometers.info/coronavirus/accessed on 26/04/2021). The clinical manifestations of coronavirus disease 2019 (COVID‐19) varied from a mild disease with fever, cough, and little or no pneumonia to a moderate disease which presented as dyspnoea, hypoxia, and pneumonia, to a critical disease characterized by systemic shock, respiratory and multiorgan failure.^4^

SARS‐CoV‐2 is transmitted through the respiratory route. The initial site of infection for the virus is the respiratory epithelium. After infecting the lung epithelium, the inflammation spills over into the circulation generating an immune response mediated by leukocytes and cellular mediators of innate immunity including monocytes, macrophages, and dendritic cells. Indeed, SARS‐CoV‐2 may infect the circulating monocytes and blood‐derived macrophages.^5^ The infected monocytes differentiate into macrophages with a greater expression of chemokine receptors and traffic via chemoattractants to the target organ. Stimulation of these receptors induces the expression of proinflammatory cytokines. Most of the COVID‐19 patients exhibit lymphopenia and pneumonia with higher levels of cytokines in the moderate disease indicating that the virus‐induced exacerbated immune responses play a key role in the pathogenesis, morbidity, mortality and recovery in patients. Further, T cells activation and excessive elimination of regulatory T cells add to uncontrolled pathogenesis in COVID‐19.^6^, ^7^

Many clinical studies have observed benefits with, intravenous Remdesivir, a combination of Lopinavir and Ritonavir, and also with convalescent plasma therapy^8^, ^9^, ^10^ for treating COVID‐19 patients. Convalescent plasma therapy is considered very beneficial initially and still being considered by many groups.^11^, ^12^, ^13^ However, still, there is a key question about durability and longevity of immunity in recovered patients. Therefore, our aim in this study was to analyse immune markers in COVID‐19 patients with mild to moderate disease and in recovered patients who had recovered from the disease and considered for convalescent plasma donations.

Here we described a novel observation that increased activated monocytes, T cells, and low A proliferation‐inducing ligand (APRIL) and B‐cell activating factor receptor (BAFFR) expressing B cells are present in COVID‐19 patients and also have sustained existence of these markers in convalescent plasma (COPLA) donors even after 3 weeks of recovery.

## 2 MATERIALS AND METHODS

### 2.1 Patients and subjects

We prospectively recruited SARS‐CoV‐2 positive patients (*n* = 34) by real‐time PCR assay with mild to moderate symptoms in the first week of disease at the Department of Internal Medicine, Lok Nayak Hospital, a designated COVID‐19 treatment center in New Delhi, India and COVID‐19 recovered patients (*n* = 15) who have recovered from the mild disease and considered for convalescent plasma donation after 3 weeks of their recovery, at Institute of Liver and Biliary Sciences (ILBS), New Delhi, India, from May to July 2020. The severity of disease was characterized by respiratory distress, respiratory rate (RR) ≥30/min, oxygen saturation level <93% in resting state, the partial pressure of oxygen (PaO_2_)/oxygen concentration (fraction of inspired oxygen [FiO_2_]) ≤300 mmHg, within 24–48 h (Table 1). Patients less than 18 years or more than 65 years of age, those with comorbid conditions (cardiopulmonary disease, structural or valvular heart disease, coronary artery disease, chronic obstructive pulmonary disease, chronic liver disease, chronic kidney disease), patients presenting with multiorgan failure, pregnant females, individuals with HIV, viral hepatitis, cancer, morbid obesity with a body mass index (BMI) more than 35 kg/m^2^, extremely moribund patients with an expected life expectancy of less than 24 h or failure to obtain informed consent were excluded from this study.

**Table 1:** Baseline characteristics of COVID‐19 patients, COPLA donors, and healthy subjects

Baseline parameters	COVID‐19 patients (*n* = 34)	COPLA donors (*n* = 15)	Healthy subjects (*n* = 10)
Mean age (year ±* SD*)	48.21 ± 9.79	38.07 ± 7.7	48.14 ± 9.05
Male (*n*, %)	22 (75.86%)	15 (100%)	7 (74%)
Chest X‐ray changes (*n*, %)	25 (86.20)	–	–
BMI (mean and *SD*)	26.31 ± 2.29	26.84 ± 2.63	26.28 ± 2.52
Respiratory rate/min (mean and *SD*)	34.5 ± 2.55	14.14 ± 2.53	14 ± 2.65
PaO_2_ (mmHg)	61.76 ± 4.96	–	–
O_2_ Saturation (%)	85.03 ± 4.03	99 ± 1	99 ± 1
FiO_2_ (mmHg)	0.38 ± 0.04	–	–
PaO_2_/FiO_2_ ratio	162.92 ± 13.77	–	–
Baseline neutrophils (N, median and range)	3375 (2626, 6928)	4328 (2789, 6100)	4535 (3160, 6000)
Baseline lymphocytes (L median and range)	968 (758, 1874)	2371 (1400–2600)	2453 (1550, 2890)
N/L ratio	3.8 (3.37, 4.67)	1.81 (1.6, 2.0)	1.67 (1.52, 1.84)
Platelet count (in lakh) (cmm^3^)	1.8 ± 1.20	1.6 ± 1.43	2.2 ± 2.42
SOFA score	7.44 ± 1.86	–	–
Baseline Ct value of RT‐PCR	31.91 ± 3.44	‐ve	–

Abbreviations: COVID‐19, coronavirus disease 2019; COPLA, considered for convalescent plasma; FiO_2_, fraction of inspired oxygen; PaO_2_, partial pressure of oxygen; RT‐PCR, reverse transcription‐polymerase chain reaction; SOFA, sequential organ failure assessment.

Recovered patients were symptom‐free for 3 weeks and had a negative real‐time PCR assay. To compare the intensity of immune markers, healthy subjects (*n* = 10) were enrolled in this study.

### 2.2 Sample collection

Both nasopharyngeal and oropharyngeal swabs were taken in a 3 ml viral transport media. A total of 6–8 ml peripheral blood was collected in ethylenediaminetetraacetic acid coated vacutainers from COVID‐19 patients at the time of admission and recovered patients after 14 days of their recovery at the time of convalescent plasma donations. Plasma was separated and stored at −80°C for further use.

### 2.3 Reverse transcription‐polymerase chain reaction (RT‐PCR)

A volume of 200 µl of the sample was processed for viral nucleic acid extraction by Qia symphony DSP Virus/Pathogen mini kit (Qiagen GmbH) as per the manufacturer's protocol. The extracted elutes of RNA was subjected to RT‐PCR for the qualitative detection of both E as well as ORF 1ab (RdRP) genes of the SARS‐CoV‐2 virus using a commercial RT‐PCR kit (nCoV RT–PCR; SD Biosensor) as per the kit literature. The sample was considered positive when fluorescence was seen in both the target genes E as well as RdRP up to a cycle threshold (Ct) value 36. Ct values were utilized as a marker to monitor the clinical progress of the patients. Two consecutive negative test results of real‐time RT‐PCR were performed 24 h apart, from combined oral and nasopharyngeal swab for donation consideration.

### 2.4 Demographic data and laboratory parameters

We collected demographic data, symptoms, and clinical signs from the medical records. Laboratory tests were conducted for serum proteins, complete blood count, transfusion‐transmitted infections (hepatitis B virus, hepatitis C virus, HIV, malaria, and syphilis), blood grouping, and immunoglobulin G antibody against SARS‐CoV‐2.

### 2.5 Analysis of plasma analytes using bead assay

The concentrations of 21 plasma cytokines, chemokine and growth factors such as interleukin (IL)‐1β, IL‐2, IL‐18, IL‐10, interferon‐γ (IFN‐γ), IL‐6, transforming growth factor beta‐1, macrophage migration inhibitory factor (MIF), macrophage inflammatory protein (MIP)‐1α, MIP‐1β, MIP‐3α, ITAC, monocyte chemotactic protein‐1 (MCP‐1), Fractalkine, ENA78, IP‐10, MCSF, Leptin, vascular endothelial growth factor (VEGF)‐A, matrix metalloproteinase 12 (MMP12) and E‐selectin were determined in 25–30 μl of plasma in all three groups using multiplexed procataplex cytokine bead assay (Thermo Fisher Scientific) following the complete details on Luminex xponent 3.1TM Rev.2 (Luminex Corporation). A standard curve was drawn using the standards provided in the kits. Values in samples were determined corresponding to the standard curve drawn. The lower limits of detection of the test for each cytokine is given in the Table S1.

### 2.6 Multiparametric whole blood immunophenotyping

Monocytes, T‐cells, B cells, and Tregs were characterized in whole blood. A total of 100 µl of whole blood sample was stored in 700 µl of FACS Lysing solution (BD Pharmingen) and was stored in −80°C for further use. Using specific antibodies against surface and intracellular markers labeled with different fluorochromes, blood mononuclear cells were characterized.

All samples were processed on a single day in one batch to avoid any batch‐to‐batch processing variation. FACS Lysed samples were thawed in 37°C water bath for a minute and immediately centrifuged and washed twice with 1X phosphate buffered saline (PBS) containing 0.5% bovine serum albumin with 0.1% Sodium Azide at 400*g* for 3 min at the room temperature. After two washes, the cell pellets were stained for surface expressing markers by incubating with appropriate cell‐specific antibodies for 30 min at room temperature in the dark. After incubation, the cells were washed and spun at 400*g* for 3 min to discard the supernatant. The collected pellets were permeabilized with 200 µl FACS permeabilization solution (BD Pharmingen) and incubated for 10 min before washing. After centrifugation, the collected cell pellets were further incubated for 30 min with specific antibodies for intracellular expression of markers. Antibodies were labeled with different fluorochromes and used for detection of B cells; anti‐CD3, CD14, CD56, CD19, CD27, CD21, IgD, CD40, CD38, CD274, CD185, CD268, BAFFR, and APRIL, monocytes; anti‐CD3, CD56, CD19, CD14, CD16, HLA‐DR, CD11b, CD11c, CCR2, CX3CR1, CD80, CD124, CD45, CD163, CD71, CD206, CD123, CD68, and T cells and their subsets; anti‐CD3, CD4, CD25, CD27, CCR7, FOXP3, GATA3, CD194, CD278, CD45RA, CD127. Finally, the cell pellets were acquired on FACS ARIA analyzer cum sorter using a minimum of 100,000 events. The data were analyzed by FlowJo software version 10.2 (BD).

### 2.7 T cells functionality assay

To investigate the CD4^+^ and CD8^+^ T cell functionality in COVID‐19 positive patients, their peripheral blood mononuclear cells were cultured in 96‐well plates in RPMI media containing 10% fetal bovine serum, with or without phorbol 12‐myristate 13‐acetate (PMA)/Ionomycin (PMA 2 ng/ml; ionomycin 1 μg/ml; Merck) and 10 µg/ml lipopolysaccharides (LPS) (Merck) at 37°C on the presence of 5% CO_2_ for 6 h. After 1 h of incubation, 1 μg/ml brefeldin A (BD Pharmingen) was added to all the wells. After incubation, the cells were surface stained for 25 min with anti‐CD3‐FITC, anti‐CD8‐BV421 and anti‐CD4‐PE/cyanine 5.5 (Cy5) followed by 10 min permeabilization with 100 µl of permeabilizing solution (BD Biosciences) and washed twice with 500 µl 1X cytoperm. The cells were then stained for the intracellular markers IL‐2, IFN‐ γ, and IL‐17 with anti‐IL2 APC, anti‐IFN‐γ PE, anti‐IL‐17 PE‐Cy7 (BD Biosciences and Pharmingen), incubated for another 25 min. Followed by washing with PBS and fixing in 0.1% para formaldehyde, the cells were analyzed on a BD Verse flow cytometer. Data were analyzed using FlowJo.

### 2.8 Plasma proteomic analysis

Total proteins were isolated from the plasma of COVID‐19 (*n* = 15) and convalescent plasma donors (*n* = 14) and were subjected to mass spectrometry analysis. In brief, total protein was extracted using radioimmunoprecipitation assay (Thermo Fisher Scientific™) as per the instructions recommended by the manufacturer. The extracted proteins were subjected to albumin depletion using ÄKTA pure (Cytiva). Proteins were then precipitated with 100% methanol overnight at −20°C, dissolved in 50 mM ammonium bicarbonate and reduced by dithiothreitol (DTT) for 40 min at 56°C. Proteins were then alkylated by 20 mM iodoacetamide (IAA) for 40 min at room temperature in the dark. The 20 μg of protein samples were then kept for overnight digestion with 1 μg sequencing grade trypsin (Promega) at 37°C. The reaction was stopped by acidifying the peptides using 0.1% formic acid followed by desalted on reversed‐phase C18 (Thermo Fisher Scientific). The peptides were then eluted using 50 µl of 60% acetonitrile in 0.1% formic acid and concentrated in a speed vac machine. The peptides were finally acidified with 2% acetonitrile, 0.1% trifluoroacetic acid in 0.1% formic acid. The samples were analyzed by nano‐LC‐MS/MS coupled to Q‐exactive TM plus mass spectrometer (Thermo Fisher Scientific) using software version 10.

### 2.9 Pathway enrichment and immune cell abundance prediction

Differentially expressed proteins (DEPs) were identified followed by the statistical analysis in metaboanalyst.^14^ The same web tool was used for performing a classical univariate receiver operating characteristic (ROC) curve and random forest analysis. The DEPs were annotated for Gene Ontology (GO) (Biological Processes) classification using GO database^15^ and predicted for hallmark gene sets using Gene Set Enrichment Analysis (GSEA).^16^ The abundance of immune cells was predicted using ImmuCellAI.^17^ The correlation network of upregulated proteins in COVID‐19 patients drew by Immune‐Navigator using Edge correlation threshold 0.41 and Significance correlation threshold 0.49.^18^

### 2.10 Statistical analysis

Continuous variables were expressed as mean or median and compared by student *t* test or Mann–Whitney *U* test as appropriate. The categorical data were analyzed using *χ* ^2^ or Fisher's exact test. To compare pre‐and postvalues, a paired *t* test or Wilcoxon signed‐rank test was used. The correlation among clinical parameters and immune cells were analyzed using the Pearson correlation test.

## 3 RESULTS

### 3.1 Plasma profile of COVID‐19 patients and recovered patients (COPLA donors)

**Figure 1:**
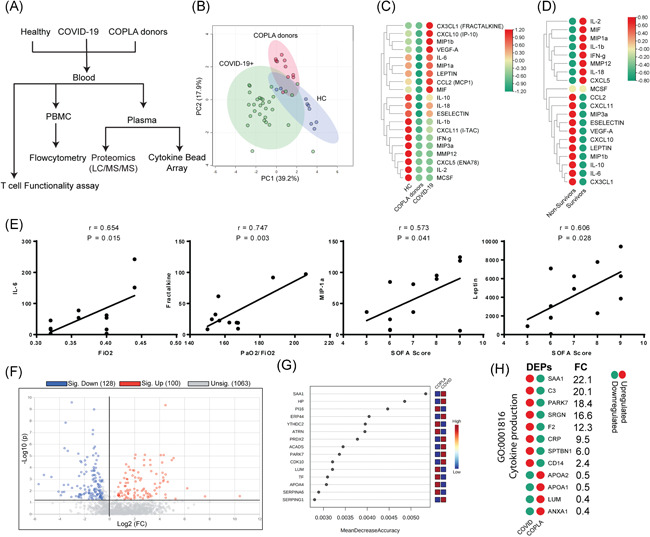
Differential expression of plasma analytes in COVID‐19 patients, COPLA donors and healthy subjects. (A) The flowchart depicts the overview of the study design. (B) The variation in the level of plasma analysts among the groups determined by the cytokine bead array was shown by principal component analysis (PCA). The results of the cytokine bead array revealed a differential cytokine level among. (C) Healthy, COPLA donors and COVID‐19 groups, and also between (D) survivors and nonsurvivors patients (E) The expression of IL‐6, MIP1‐α, Leptin and Fractelkine was significantly correlated with FiO_2_ level and SOFA score. (F) Differentially expressed proteins (DEPs; 1.5‐fold change) between COVID‐19 patients and COPLA donors was shown as a Volcano plot. (G) The proteins were ranked by their contributions to classification accuracy (mean decrease accuracy). (H) DEPs in the serum of COVID‐19 patients are linked with cytokine production. A *p* value of less than .05 was considered significant. COPLA, considered for convalescent plasma; COVID‐19, coronavirus disease 2019; DEP, differentially expressed protein; IL, interleukin; SOFA, sequential organ failure assessment

The recruited samples included healthy individuals, COVID‐19 patients, and COPLA donors; patients who recovered and donated their convalescent plasma. The plasma and serum samples of the groups were subjected to RT‐PCR, Cytokine Bead Array, and proteomics analysis (Figure 1A). The COVID‐19 RT‐PCR positive patients had a moderate disease with a high respiratory rate (34.47 ± 2.5), low O_2_ saturation (85 ± 4.03), low baseline PaO_2_/FiO_2_ ratio (162.92 ± 13.77), and X‐ray changes. Recovered patients who were considered for convalescent plasma donation had a history of mild infection, were RT‐PCR negative and at least 14 days passed from their recovery. The mean age, gender, and BMI were comparable between the groups (Table 1).

Principal component analysis revealed a differential level of serum analytes in the serum of the groups (Figure 1B). CX3CL1, CXCL10, MIP1b, VEGF‐A, IL‐6, MIP1a, Leptin, CCL2, and MIF were significantly increased in COVID‐19 positive patients compared to the COPLA donors and healthy subjects. These analytes in the COPLA donors were decreased after 15–21 days of recovery. In addition, a downregulated level of IL‐1β, E‐selectin, CXCL11, IFN‐γ, MIP3a, MMP12, CXCL5, IL‐2, and MCSF was found in the COVID‐19 positive patients (Figure 1C). A significant increase in the level of CCL2, CXCL11, MIP3a, E‐selectin, VEGF‐A, CXCL10, Leptin, MIP1b, IL‐10, IL‐6, and CXCL1 were detected in the non‐survivors compared to the survivors (Figure 1D). Furthermore, the correlation analysis showed that few serum analytes were significantly correlated with the clinical parameters in the COVID‐19 patients. An increase in IL‐6, MIP1b, and Leptin and CXC3L1 (Fractalkine) was significantly correlated with the enhanced FiO_2_ concentration and sequential organ failure assessment (SOFA) score (Figure 1E).

Additionally, the serum proteins of COVID‐19 patients and COPLA donors were analyzed by LC‐MS/MS (Figure 1F and Figure S1A,B). Out of 1291 identified proteins (Table S1), 100 proteins were upregulated and 128 were significantly downregulated in COVID‐19 patients compared to the COPLA donors (Figure 1G). Among the upregulated proteins, 19 proteins were previously shown to be associated with the SARS‐CoV‐2 infection (Figure S1C). Performing the classical univariate ROC curve analyses (Figure S1D) and the random forest analysis (Figure 1G) showed that SAA1 (Serum amyloid A1), SERPING1 (Plasma protease C1 inhibitor), and SERPING3 were potential markers associated with the SARS‐CoV‐2 infection. Furthermore, the GO analysis of DEPs showed that expression level of cytokine production associated (GO:0001816) proteins like SAA1, C3, PARK7, SRGN, F2, and CRP was enhanced more than 10‐fold in COVID‐19 patients (Figure 1H).

### 3.2 Increased CXCR3^+ve^ and CCR2^+ve^ monocytes in COVID‐19 positive patients and COPLA donors

**Figure 2:**
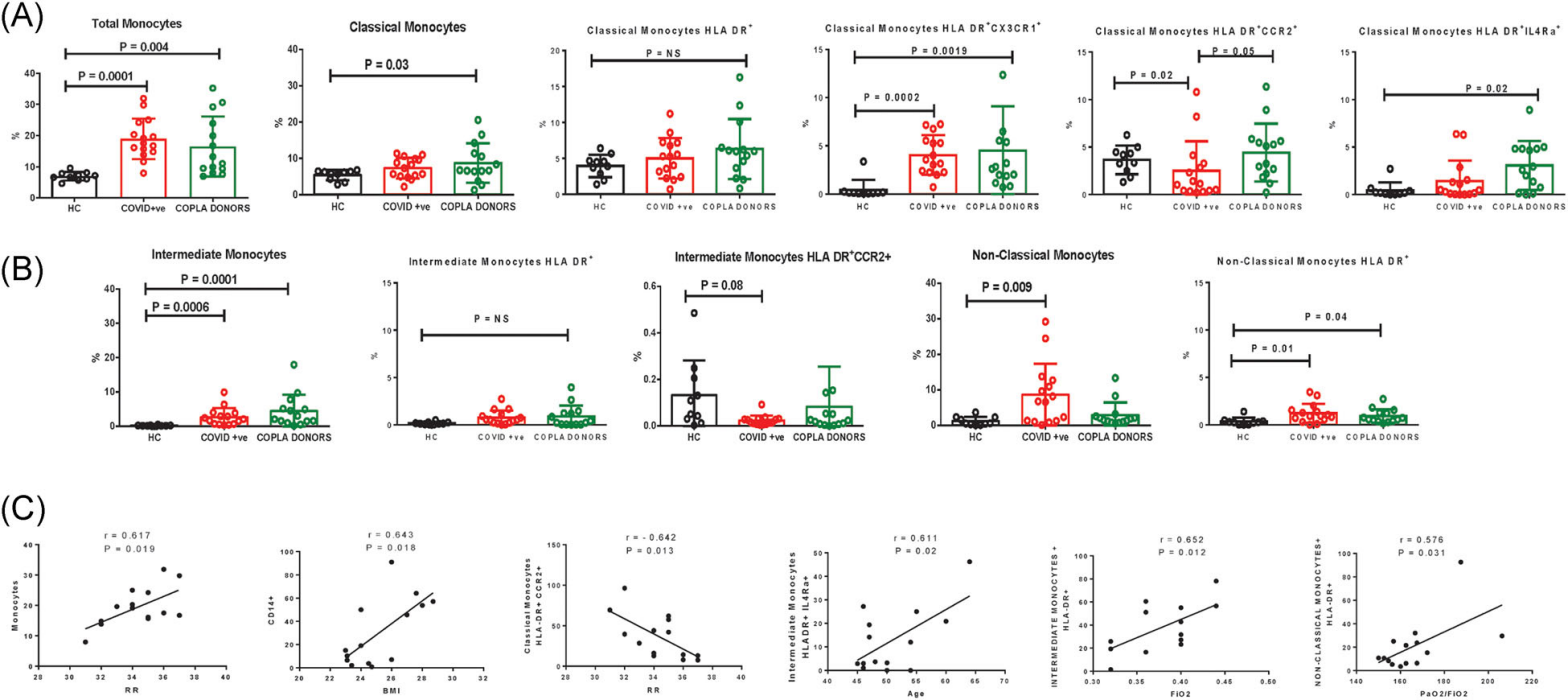
Total monocytes and their subset population in moderate COVID‐19 patients recovered COPLA donors and healthy subjects. The flow cytometry analysis showed (A) the increase in the total monocytes in COVID‐19 and COPLA donors with enhanced expression of HLA‐DR and CX3CR1 in classical monocytes. (B) The intermediate monocytes in COVID‐19 and COPLA donors increased compared to the healthy subjects but with low expression of CCR2. COVID‐19 patients showed increased nonclassical monocytes and HLA‐DR expression. (C) The changes in the monocytes markers were correlated with the clinical parameters. Error short bars show the mean ± *SEM. p* < .05 was considered significant. BMI, body mass index; COPLA, considered for convalescent plasma; COVID‐19, coronavirus disease 2019; r, correlation coefficient; rr, respiratory rate

The whole blood was analyzed by flow cytometry to characterize their immune profile. The gating strategies of the monocytes is given in supplementary material (Figure S2). The total circulating monocytes were increased in the moderate patients and the recovered COPLA donors compared to the healthy controls (Figure 2A). Further analysis showed that there was no significant change in the number of classical monocytes (CD14^++^16^−^) in the COVID‐19 patients compared to healthy controls; however, an increase in the intermediate (CD14^++^16^+^) and nonclassical (CD14^+^CD16^++^) subsets were observed (Figure 2A,B). Despite no change in the number of classical monocytes, the dual expression of HLA‐DR/CCR2 and HLA‐DR/CX3CR1 was increased in the COVID‐19 patients. However, the CCR2 expression was reduced in the intermediate subset in COVID‐19 patients.

In the COPLA donors, we observed that the classical monocyte compartment was significantly increased with no change in HLA‐DR but with sustained expression of CX3CR1 (Figure 2A,B). Furthermore, the expression of HLA DR^+^IL4Ra^+^ was significantly more in COPLA donors (Figure 2A). We further identified that in the COVID‐19 patients, an increase in the total monocytes was significantly correlated with the increase in the BMI and RRs. However, a decreased CCR2 expression was correlated with the higher RR in COVID‐19 patients, the increased HLA‐DR on intermediate and nonclassical monocytes and IL4RA expression was correlated with increased age, FiO_2_ and PaO_2_/FIO_2_ levels (Figure 2C).

### 3.3 Increased central memory T cells in COVID‐19 patients and COPLA donors

**Figure 3:**
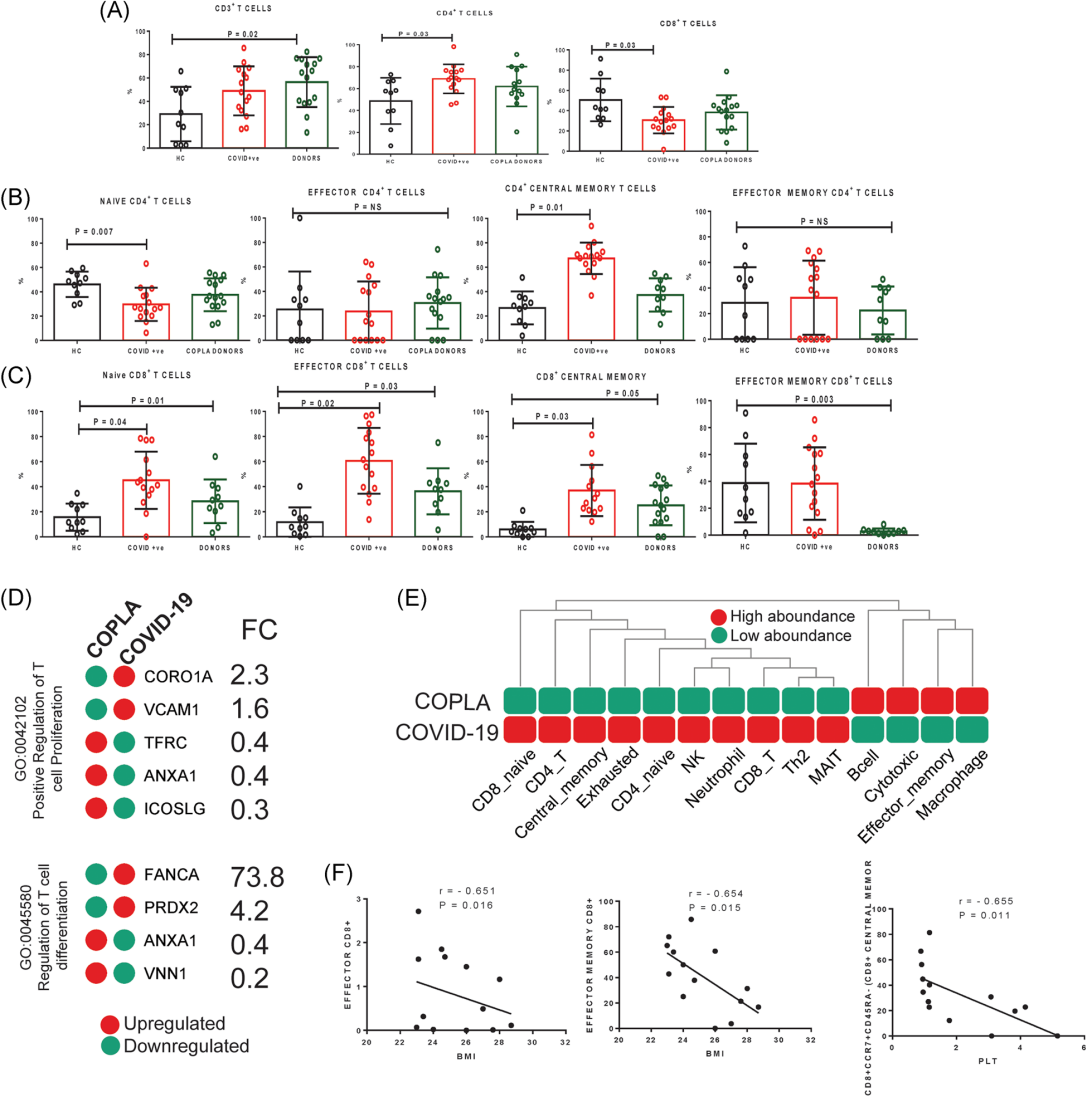
Total T cells and their subsets in the moderate COVID‐19 patients, COPLA donors and healthy subjects. (A) The percentage of circulating CD3^+^, CD4^+^, and CD8^+^ T cell subsets were compared among the groups using flow cytometry analysis. (B and C) The CD4^+^ and CD8^+^ T cell subsets as naïve, effector, central memory and effector memory were characterized among the three groups. (D) Few differentially expressed proteins (DEPs) in the serum of the COVID‐19 patients were found to be associated with T cell proliferation and differentiation. (E) The immune cell abundance analysis using the proteomics results showed the probable immune profile of COVID‐19 patients. (F) The decreased effector and effector memory CD8^+^ T cells was significantly correlated with higher BMI and decreased central memory cells were correlated with the decrease in platelets in moderate COVID‐19 patients. Error short bars show the mean ± *SEM*. *p* < .05 was considered significant. BMI, body mass index; COPLA, considered for convalescent plasma; COVID‐19, coronavirus disease 2019; PLT, platelet count; r, correlation coefficient; rr, respiratory rate

In patients, the CD3^+^ T cell compartment was not significantly different from the healthy subjects; however, the ratio of CD4^+^/CD8^+^ T cells was skewed in patients more towards CD4^+^ T cells (Figure 3A). However, the correlation network analysis of upregulated proteins in serum revealed a stronger correlation of expression in CD8^+^ cells compared to CD4^+^ cells in COVID‐19 patients (Figure S4A).

We further compartmentalized T cells, based on CD45RA, CCR7, and CD27 markers expression into naïve, effector, central memory and effector memory T cells. It was observed that in the COVID‐19 patients, CD4^+^ naïve cells were reduced but the central memory T cells were increased. However, in the CD8^+^ T cell compartment all naïve, effector, central, and effector memory subsets were increased compared to the healthy samples. Further, in COPLA donors we did not observe any change in CD4^+^/CD8^+^ T cells compared to the healthy controls; however, they still had a greater expression of effector and central memory, but reduced effector memory compartment of CD8^+^ T cells (Figure 3B,C). Besides, the proteomics analysis showed that in the COVID‐19 patients the upregulated proteins are more associated with the immune effector process (GO:0002697 and GO:0002252) than the downregulated proteins (Figure 3A,B). Further, the presence of upregulated CORO1A (2.3‐fold), VCAM1 (1.6‐fold), FANCA (73.8‐fold) and PRDX2 (4.2‐fold) COVID‐19 patients serum indicated the increased potential T cells proliferation and differentiation (Figure 3D). Immune cell abundance analysis also predicted a more abondance for central memory and exhausted cells in the patients compared to the COPLA donors (Figure 3E and Figure S4B).

**Figure 4:**
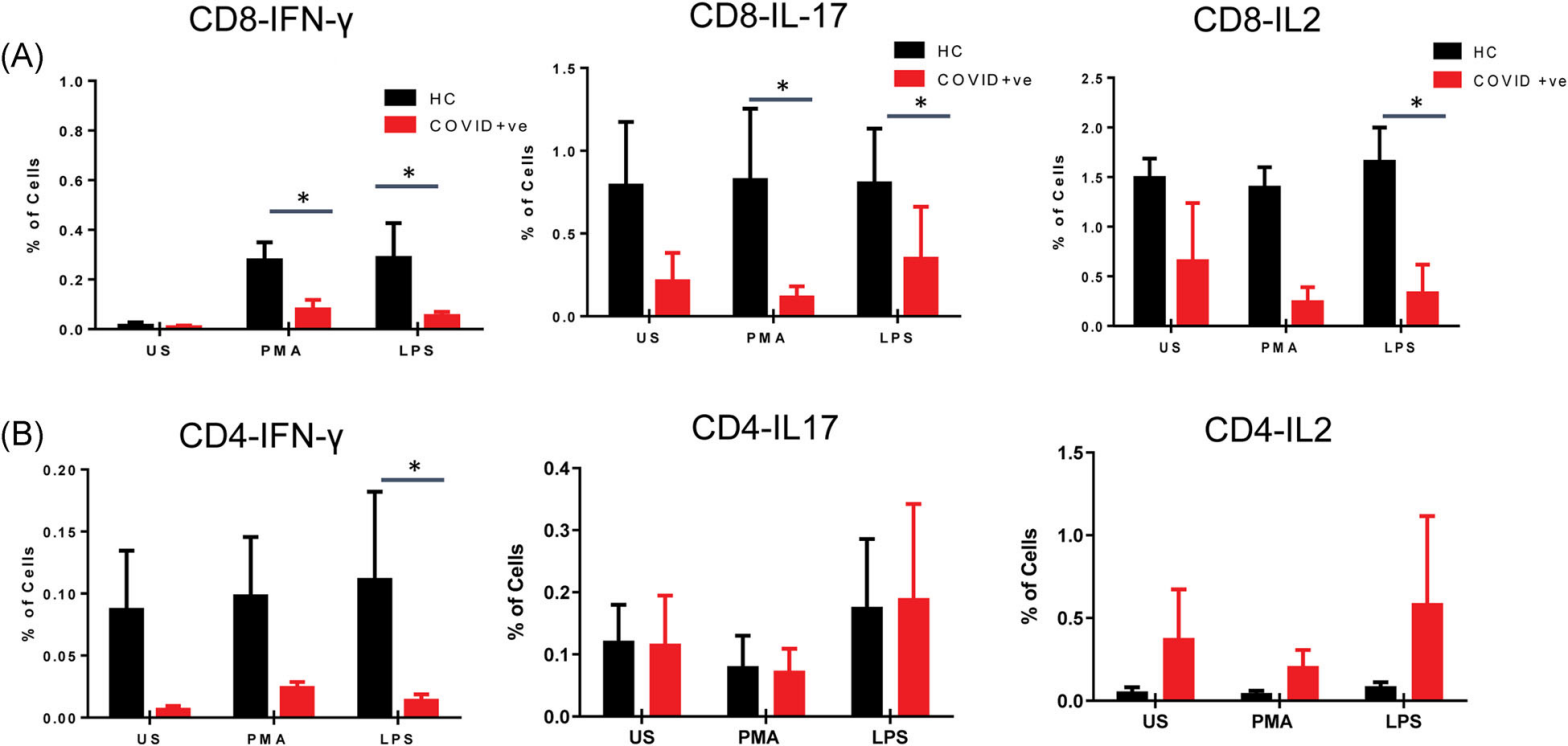
Functional assay of T cells displaying IFN‐γ, IL‐17A, and IL‐2 secretory cytokines. PBMCs were stimulated with LPS and PMA/ionomycin as positive controls for 6 h followed by phenotypic surface marker and intracellular staining. The flow cytometry analysis revealed the percentage of IFN‐γ, IL‐17A, and IL‐2 producing (A) CD8^+^ and (B) CD4^+^ T cells with or without stimulations. *p* < .05 is considered significant. IFN‐γ, interferon‐γ; IL, interleukin; LPS, lipopolysaccharides; PMA, phorbol 12‐myristate 13‐acetate; US, unstimulated

In the COVID‐19 patients, the effector, effector memory and central memory CD8^+^ T cells were negatively correlated with the increase in BMI and platelets count (Figure 3D). We have also observed that the increased effector CD8^+^ T cells but not CD4^+^ T cells in the COVID‐19 patients could secrete IFN‐γ, IL‐17 and IL‐2 upon LPS stimulation in vitro. LPS stimulated CD4^+^ and CD8^+^ T cells were less polyfunctional in the moderate COVID‐19 patients (Figure 4). Accordingly, GSEA analysis of upregulated proteins in the serum of COVID‐19 patients showed a high association with the hallmark gene sets for IFN‐γ response, IL2‐STAT5 signaling and complement (Figure S4C).

### 3.4 Downregulation of APRIL and BAFR in B cells

**Figure 5:**
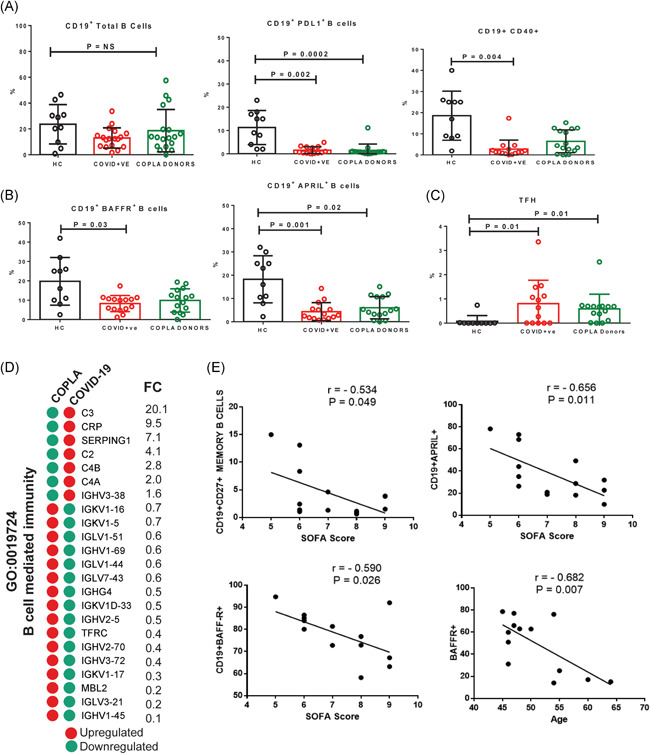
Circulating B cells, their subsets and TFH cells. (A) The percentage of circulating CD19^+^ B cells and the expression of PDL1, CD40, (B) BAFFR, and APRIL on CD19^+^ cells, and also (C) the frequency of TFH cells was determined by flow cytometry analysis. (D) The DEPs associated with the B cell‐mediated immunity were mostly downregulated in the serum of COVID‐19 patients. (E) The decreased percentage of B cells and expression of APRIL and BAFFR were correlated with the increased SOFA score and age in the moderate COVID‐19 patients. r is representing the correlation coefficient. Error short bars show the mean ±* SEM. p* < .05 was considered significant. APRIL, A proliferation‐inducing ligand; BAFFR, B‐cell activating factor receptor; COVID‐19, coronavirus disease 2019; DEP, differentially expressed protein; TFH, T follicular helper

A reduction in the total B cells was observed in the COVID‐19 patients; however, it was not significantly less than the healthy subjects (Figure 5A). However, the activated B cells were decreased with a lower expression of PDL1 and CD40. Indeed, it was observed that BAFFR were significantly reduced in the patients, while the APRIL expressing B cells were significantly reduced in both patients and COPLA donors (Figure 5B). The T follicular helper cells were significantly enhanced in both groups (Figure 5C). In addition, the proteomics analysis showed that among DEPs, 16 proteins that were associated with B cell‐mediated immunity (GO:0019724), were downregulated in the serum of COVID‐19 patients (Figure 5D). The correlation analysis also revealed that the decreased memory B cells, APRIL and BAFFR expression was negatively correlated with the SOFA score and age of the COVID‐19 patients (Figure 5E).

## 4 DISCUSSION

Analysis of plasma analytes using the cytokine bead assay revealed the involvement of inflammatory monocytes by raising the levels of MIPs, MIF, and CCL2 (MCP‐1) in the COVID‐19 patients compared to the healthy controls and COPLA donors. Indeed, most of the proinflammatory cytokines such as IL‐2, IL‐1b, IFN‐γ, were expressed similar or lower than the range in healthy controls. Only four patients who did not survive for a week had marginally increased IL‐6 levels along with MIPs and MIF. This analysis suggests that other than reported IL‐6 and TNF‐α, other proinflammatory cytokine storms also do play a role in the severity of COVID‐19. Earlier findings also showed high levels of IP‐10, MCP‐1, MIP‐1A, in most of the patients with moderate COVID‐19.^19^ It was observed that instead of an increase in the typical proinflammatory cytokine storm, dynamic immune responses of COVID‐19 patients exhibited the role of IL‐1 signaling in moderate cases.^20^ We have also observed that IL‐6 levels were significantly correlated with FiO_2_. Furthermore, COVID‐19 were more moderate in the elderly and subjects with comorbidities but also having the scope of blunted immunity in the elderly may reflect hypo responsiveness for cytokine storm.

The proteomics analysis highlighted the role of SAA1 and SERPING1 in the COVID‐19 patients. SAA1 is one of the most important biomarkers in acute inflammation. Enhanced SAA1 level is correlated with the increased immune infiltrating cells.^21^ Besides, it has been shown that SAA1 may serve as a biomarker of poor prognosis for COVID‐19 as the patients with higher initial SAA levels were shown to be more likely to have poor CT imaging.^22^ Besides, the increased level of SERPING1 which transcriptionally regulated by IFN‐γ was shown to be correlated with COVID‐19. It was suggested that as SERPING1 works as in a negative feedback loop to control contact and complement system activation, the enhanced level of SERPING1 is an attempt by the host to constrain inappropriate complement activation.^23^

SARS‐CoV‐2 may primarily infect monocyte and CD14^+^ subsets with higher expressions of CCR2 and CX3CR1 which may differentiate and migrate from the periphery to the target sites as macrophages during infection.^24^ Monocytes with higher expression of CCR2 are also considered as senescent monocytes causing inflammatory disease in the elderly due to their shortened telomeres.^25^ Although convalescent plasma therapy significantly reduced the RR, improved O_2_ saturation, and improved the PaO_2_/FiO_2_ ratio in COVID‐19 patients; COPLA donors had no significant decline in monocyte activation and differentiation. This suggests that patients who have recovered and considered for COPLA donations still have compromised immunity with sustained expression of inflammatory monocytes and activated T cells. The effects of these inflammatory cytokines should be observed postinfusion into plasma recipients suffering from COVID‐19, and are already in a hyper‐inflammatory state. Increased plasma MCP‐1 and CCR2 on monocytes in COVID‐19 patients and COPLA donors strongly suggest that SARS‐CoV‐2 specifically target this population and is responsible for keeping a sustained inflammatory environment even after clearance of this virus.^26^

In viral infections, although the functional role of CD8^+^ T cell is crucial still needs to be well‐modulated in order not to cause moderate pathology. But T cell responses especially CD8^+^ T cells in SARS‐CoV‐2 infection showed greater magnitude in the secretion of IFN‐γ and TNF‐α than CD4^+^ T cells. The early rise of CD8^+^ T cells and IL‐4, IL‐5, and IL‐10 producing TH2 cells were detected more in patients with mortality. The present study revealed a bigger pool of CD8^+^ T cells secreting IL‐17, IFN‐γ, and IL‐2 than CD4^+^ T cells; however, lower than healthy subjects. Although, the protective or destructive role of Th17 in human coronavirus infection remains unanswered.^19^ Indeed, in the convalescent phase, we observed Th1 type helper T cells, but still, there was a low expression of activation markers on B cells. APRIL and BAFFR expression regulate the compartment of translational B cells to mature B cells. Recently, APRIL and BAFF expression was also observed more in IL‐10 producing regulatory B cell compartment. Bregs acts as copartners with Tregs in inhibiting excessive inflammation. Although, Bregs skew T cells expression and also are functional in suppressing Th1 and Th17 differentiation, in our study, we have observed low expression of BAFFR and APRIL on B cells suggesting that B cells were not actively proliferating, although in COPLA donors their expression was enhanced compared to the COVID patients, still it was under‐expressed than healthy.

In conclusion, SARS CoV‐2 infection induces pathogenesis by monocyte trafficking and differentiation via CCR2 and CX3CR1, MIPs, and MIFs and with reducing APRIL and BAFFR expression, and there was sustained expression of these markers even after 3 weeks of recovery in COPLA donors.

## CONFLICT OF INTERESTS

The authors declare that there are no conflict of interests.

## AUTHOR CONTRIBUTIONS

Meenu Bajpai, Suresh Kumar, Shiv K Sarin, and Ashish Maheshwari recruited, characterized, and conducted the clinical investigations of all patients and COPLA donors. Nirupama Trehanpati conceptualized the study, Ravinder Singh and Nirupama Trehanpati processed all samples in the laboratory. Hamed Hemati performed all the proteomics and bioinformatics analysis. Jayesh Kumar Sevak and Islam, Mojahidul conducted the cytokine bead assay. Pushpa Yadav and Sonal Agrawal, analyzed cytokine bead assay and flow cytometry data along with Nirupama Trehanpati. NTP and Hamed Hemati wrote and finalized the manuscript. Shiv K Sarin provided critical inputs.

## ETHICS STATEMENT

The study was approved by the review board and the Ethical Committee of the Institution of Liver and Biliary Sciences, New Delhi, India (No.F.37/(1)/9/ILBS/DOA/2020/20217/260). Informed consent was obtained from each patient or their legal guardians. Each patient was followed till discharge from the hospital. All investigations were performed in accordance with the declaration of Helsinki.

## Supporting information

Supplementary information.

## Data Availability

The data that support the findings of this study are available from the corresponding author upon reasonable request.
