# Supplementary material for: Sustained expression of inflammatory monocytes and activated T cells in COVID‐19 patients and recovered convalescent plasma donors
Source: Immun Inflamm Dis. 2021 Aug 6;9(4):1279–90. doi: 10.1002/iid3.476 (PMC8427128; doi:10.1002/iid3.476)
Supplement: Supplementary file 1 — Supplementary information. [file IID3-9-1279-s001.docx]

**Sustained expression of inflammatory monocytes and activated T cells in COVID-19 patients and recovered convalescent plasma donors**

**Ravinder Singh**^a, $^**, Hamed Hemati** ^a,$^**, Meenu Bajpai** ^b^**, Pushpa Yadav** ^a^**, Ashish Kumar Maheshwari** ^b^**, Suresh Kumar** ^c^**, Sonal Agrawal** ^a^**, Jayesh Kumar Sewak** ^a^**,** Mojahidul Islam^a^ ^a^**, Jaswinder Singh Mars** ^a^**, Shiv K Sarin** ^d^**, Nirupama Trehanpati**^a*^

^a^ Laboratory of Molecular Immunology, Department of Molecular and Cellular Medicine, Institute of Liver and Biliary Sciences, New Delhi, India

^b^ Department of Transfusion Medicine, Institute of Liver and Biliary Sciences, New Delhi, India

^c^ Department of Medicine, Lok Nayak Jai Prakash Hospital, New Delhi, India

^d^ Department of Hepatology, Institute of Liver and Biliary Sciences, New Delhi, India

*** Corresponding author**

Nirupama Trehanpati, M.Sc, Ph.D.

Professor in Molecular and Cellular Medicine, Institute of Liver and Biliary Sciences, D-1, Vasant Kunj, New Delhi, 110070, India

*E-mail addresses*: [trehanpati@gmail.com](mailto:trehanpati@gmail.com), [trehanpati@ilbs.in](mailto:trehanpati@ilbs.in),

**$: Both authors have equal contribution in this manuscript**

**Supplementary Table 1:** Lower limit of Cytokine levels in Cytokine Bead Assay

| **Cytokine** | **Pg/ml** |
| --- | --- |
| IL-6 | 10 |
| IL-2 | 5.71 |
| IL-10 | 1.7 |
| IFN- γ | 15 |
| MIF | 0.21 |
| MCP-1 | 3.93 |
| IP-10 | 2.03 |
| IL-1b | 1.99 |
| IL-18 | 17 |
| TGF-b1 | 3 |
| MIP-1α | 2.04 |
| MIP-1β | 5.64 |
| MIP-3α | 4.44 |
| ITAC | 3.74 |
| FRACTALKINE | 1.25 |
| ENA78 | 6.59 |
| MCSF | 14 |
| LEPTIN | 16 |
| VEGF-A | 5.37 |
| MMP12 | 5.76 |
| E-SELECTIN | 206 |

**
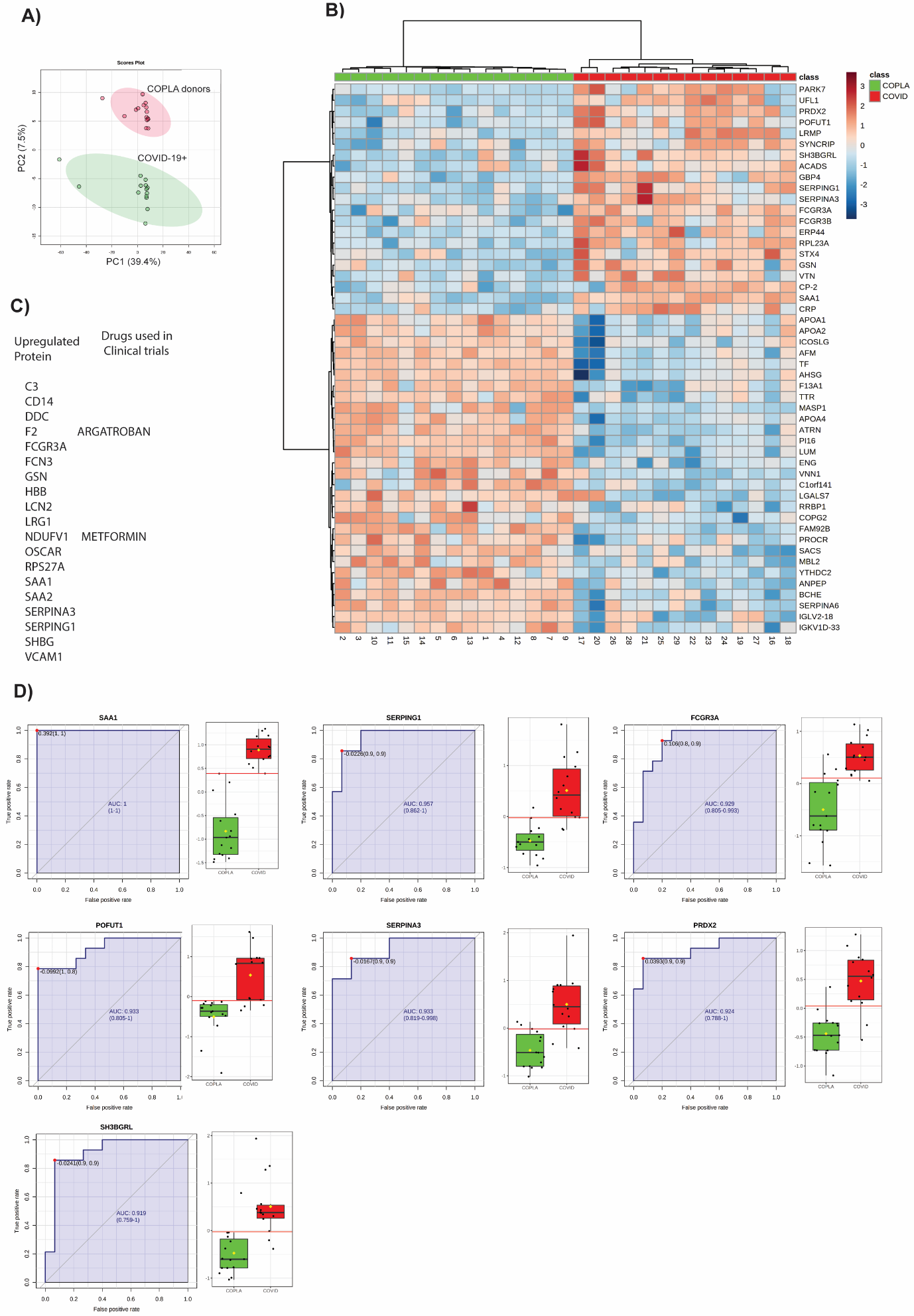
Supplementary Fig. 1.** The proteomics of the serum of COVID-19 and COPLA donors. (A) The Principal Component Analysis (PCA) shows a clear difference between the proteins in the serum of COVID-19 and COPLA donors. (B) The top 50 proteins differentially expressed among the samples were shown in the heatmap. (C) The upregulated proteins in the COVID-19 patients and their associated drugs were previously reported to be linked with SARS-coV-2 (source: [www.targetvalidation.org](http://www.targetvalidation.org)). (D) The top-ranked upregulated proteins in the COVID-19 samples based on area under the ROC curve (AUROC).


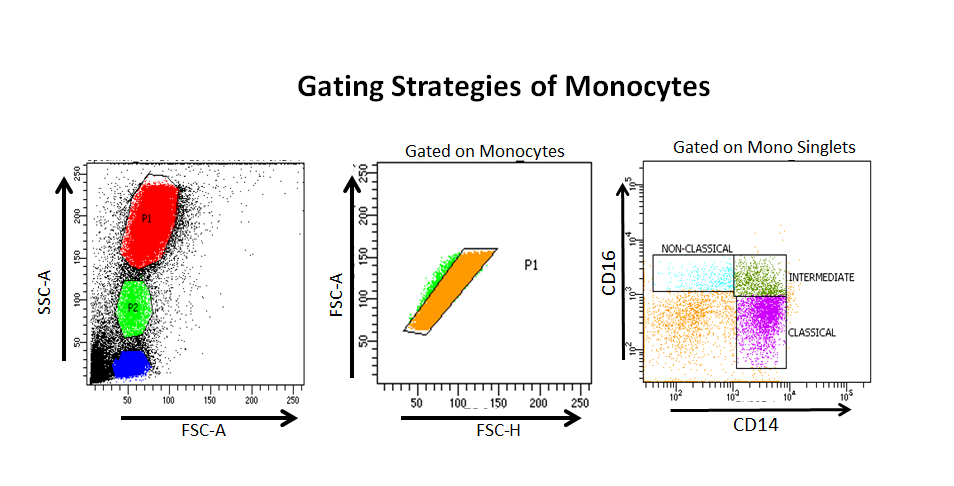


**A)**

**B)**


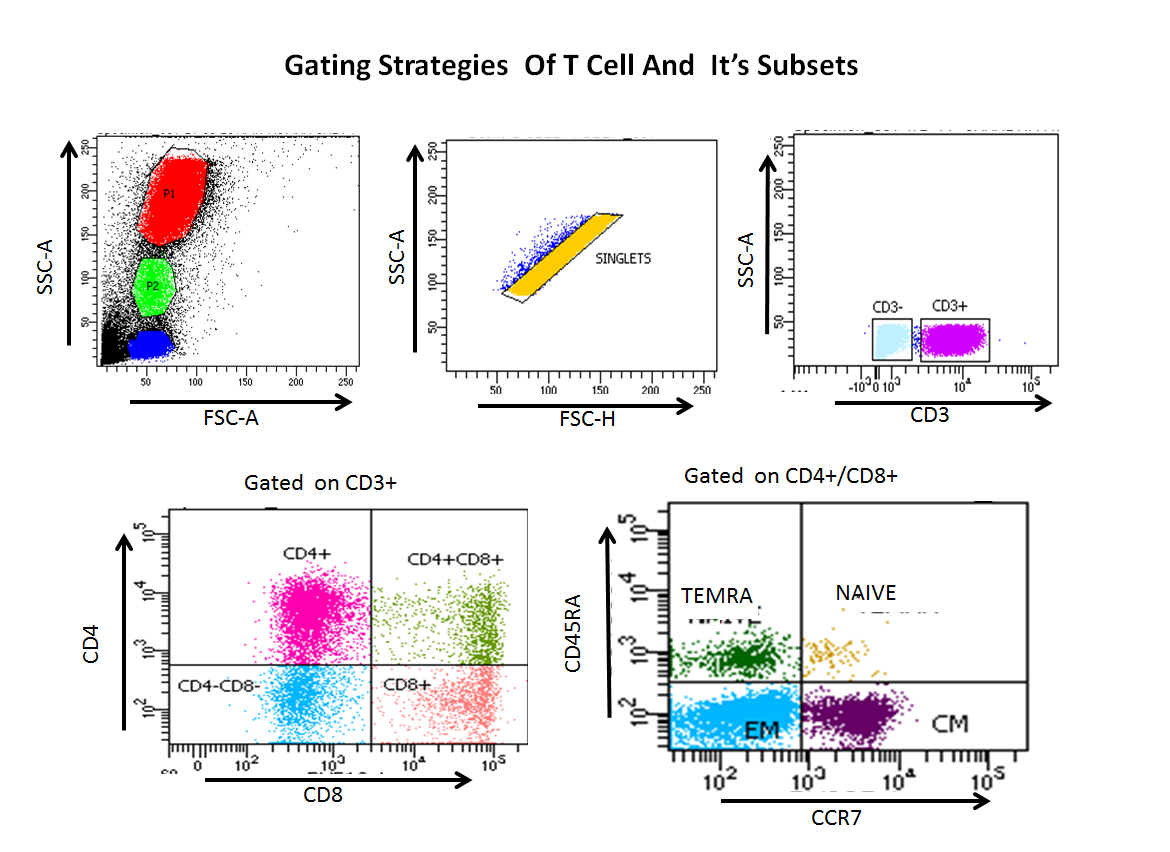


**C)**


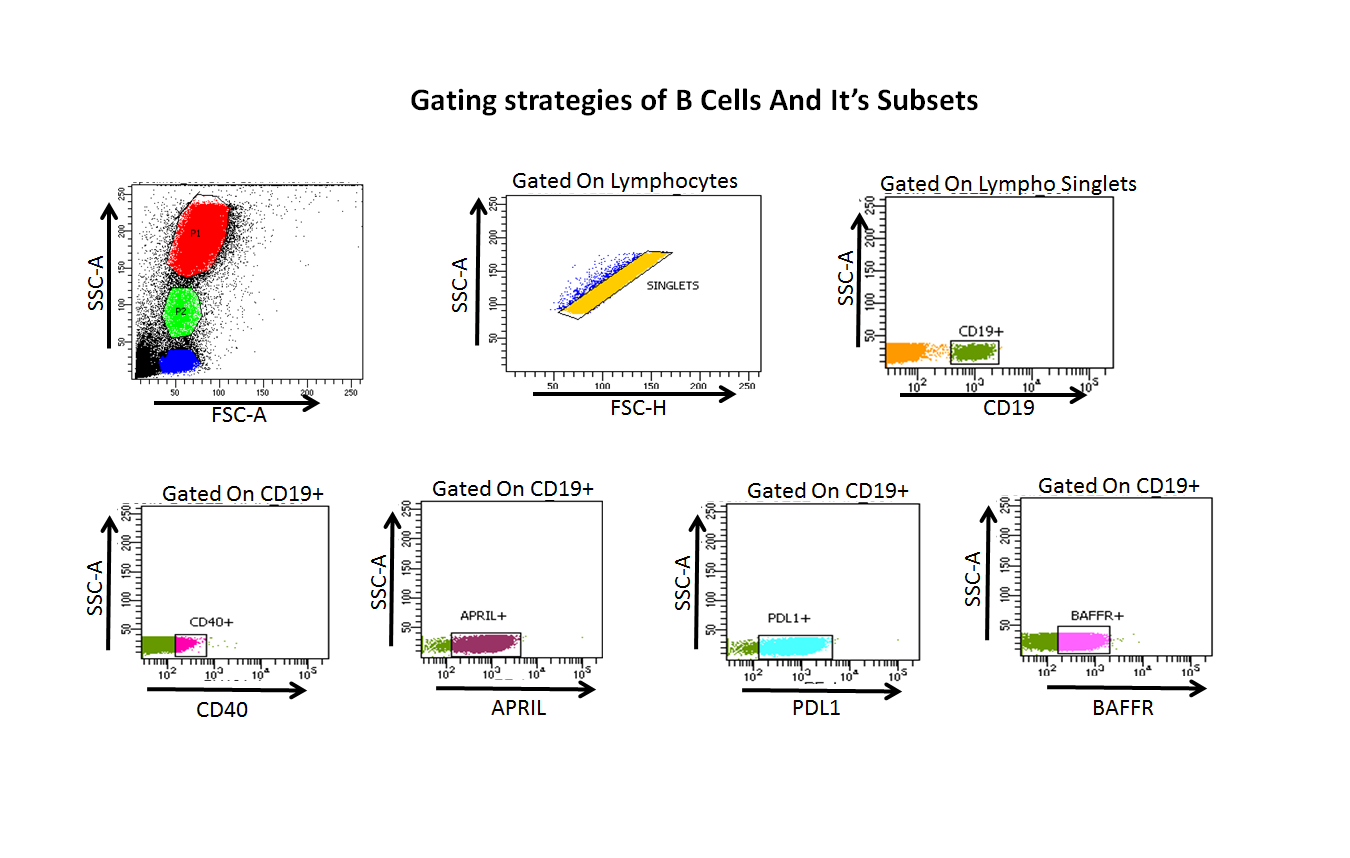


**Supplementary Fig. 2.** The gating strategies which were employed to identify the (A) monocytes, (B) T cells and (C) B cells and their subsets.


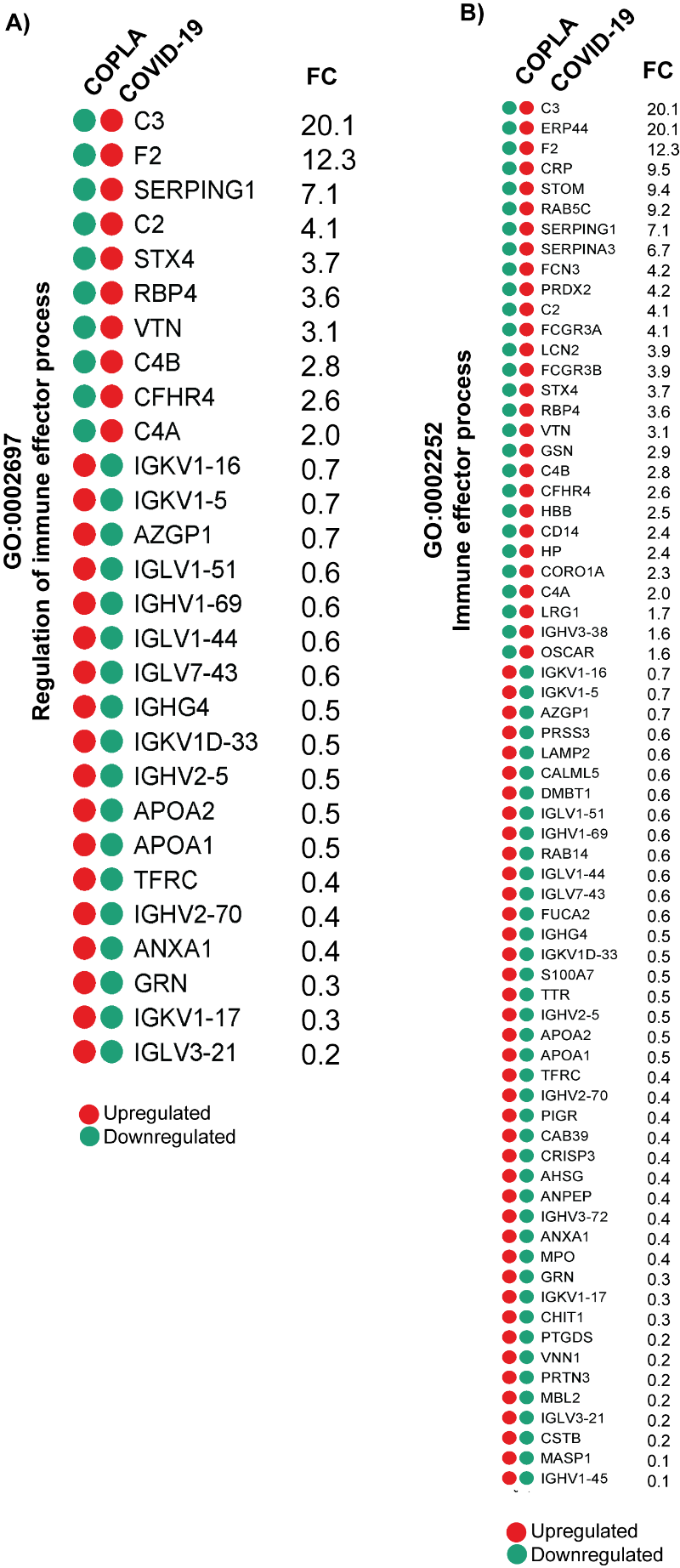


**Supplementary Fig. 3.** Gene ontology analysis of DEPs. DEPs among two COVID-19 and COPLA donor groups were highly associated with (A) the regulation of immune effector process (GO:0002697) and (B) the Immune effector process (GO:0002252).


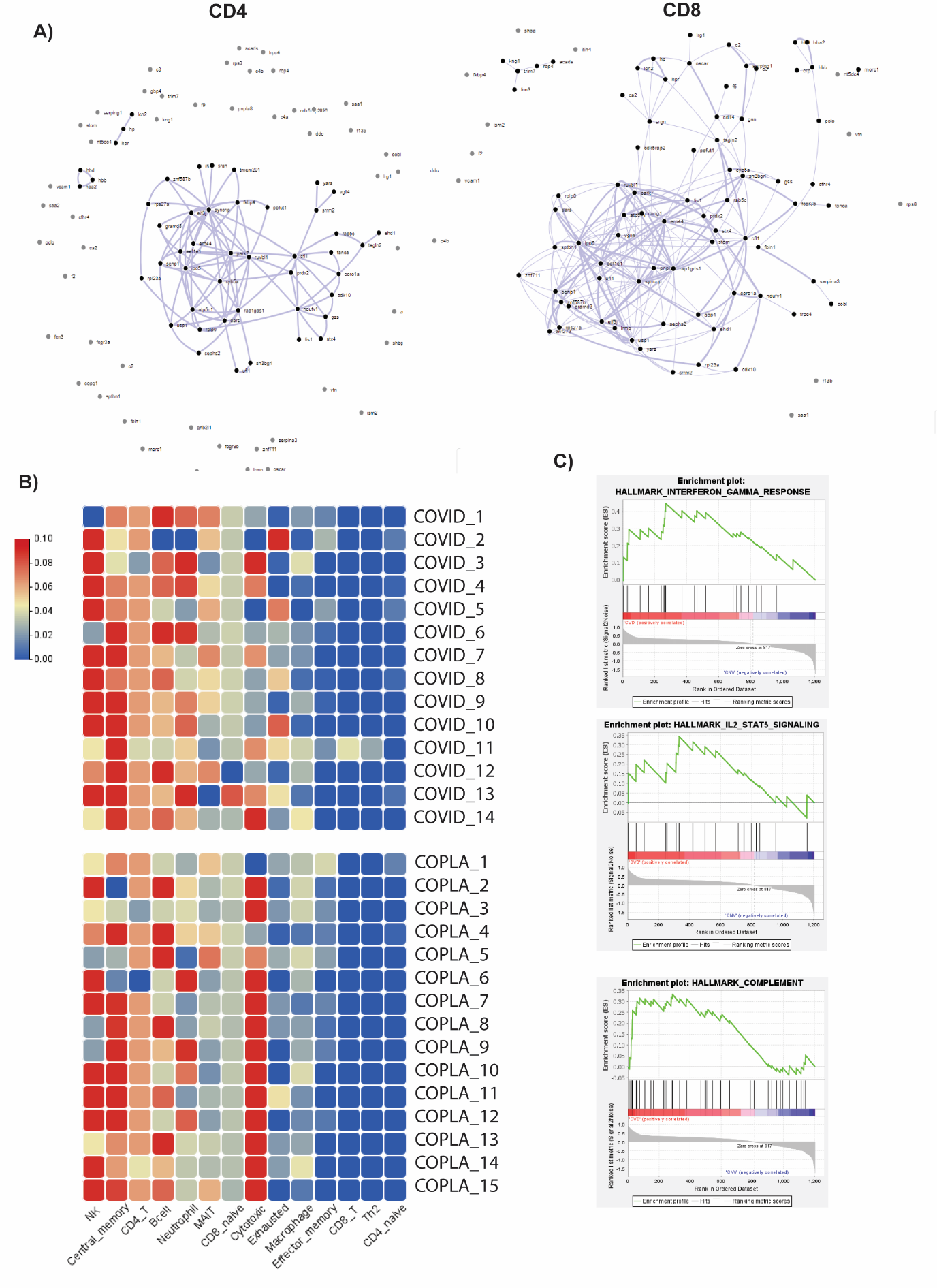


**Supplementary Fig. 4.** Bioinformatics analysis to predict immune profile of COVID-19 patients using the serum proteomics. (A) The correlation network analysis was made using the upregulated proteins to show the correlation of expression in CD8^+^ and CD4^+^ cells. (B) The immune cell abundance analysis using the serum proteomics data was performed to compare the probable abundance of immune cells among the COVID-19 patients and COPLA donors.
